# Response to correspondence on: “Neurolymphatic clearance in neurodegenerative disease: Emerging mechanisms and potential translational strategies”

**DOI:** 10.1016/j.jpra.2026.03.036

**Published:** 2026-03-27

**Authors:** Adriano Fabi, Raphael Guzman, Marc Aurel Busche, Elisabeth A. Kappos

**Affiliations:** a Department of Plastic, Reconstructive, Aesthetic and Hand Surgery, University Hospital of Basel, Spitalstrasse 21, 4031 Basel, Switzerland; b Faculty of Medicine, University of Basel, Klingelbergstrasse 61, 4056 Basel, Switzerland; c Department of Neurosurgery, University Hospital of Basel, Spitalstrasse 21, 4031 Basel, Switzerland; d Department of Biomedicine, University of Basel, Hebelstrasse 20, 4031 Basel, Switzerland; e Memory Clinic, Felix Platter University Hospital of Basel, Burgfelderstrasse 101, 4055 Basel, Switzerland

*Dear Sir,*

We thank Al Sammour and colleagues for their thoughtful correspondence regarding our review on neurolymphatic clearance in neurodegenerative diseases.^1^^,^^2^ Their perspective highlighting chronic subdural hematoma (CSDH) as a potential translational model for studying neurolymphatic modulation is both interesting and well taken.

As correctly noted by the authors, one of the main challenges in evaluating neurolymphatic interventions in neurodegenerative disorders lies in the slow and multifactorial nature of disease progression and the reliance on indirect clinical outcome measures, such as biomarker changes or cognitive scales. Detecting clinically meaningful improvements in these conditions may therefore require prolonged observation periods. In contrast, conditions such as CSDH, which are characterized by potentially reversible fluid accumulation, may offer a more accessible model to study lymphatic clearance mechanisms.^3^ However, CSDH should be viewed as a complementary model of neurolymphatic modulation rather than a surrogate for neurodegenerative disease itself, given its distinct biology and relative reversibility.

Although the primary focus of our review was on neurodegenerative diseases, the broader concept of impaired neurolymphatic clearance is likely not limited to these conditions. Increasing evidence suggests that meningeal lymphatic pathways may contribute to the regulation of intracranial fluid homeostasis and inflammatory signaling across a spectrum of neurological disorders.^4^ In this context, exploring the potential role of neurolymphatic mechanisms in the pathophysiology and resolution of CSDH is a compelling hypothesis that warrants further investigation.^5^

We appreciate the authors’ contribution to the expanding discussion surrounding neurolymphatic physiology and its potential clinical implications. Continued interdisciplinary collaboration between plastic surgery, neurosurgery, neurology and many more will be essential to further elucidate these mechanisms and their potential translational value.

## Declaration of competing interest

The authors declare that they have no competing interests.
